# Impact of *calreticulin* mutations on treatment and survival outcomes in myelofibrosis during ruxolitinib therapy

**DOI:** 10.1007/s00277-025-06204-5

**Published:** 2025-01-20

**Authors:** Francesca Palandri, Filippo Branzanti, Erika Morsia, Alessandra Dedola, Giulia Benevolo, Mario Tiribelli, Eloise Beggiato, Mirko Farina, Bruno Martino, Giovanni Caocci, Novella Pugliese, Alessia Tieghi, Monica Crugnola, Gianni Binotto, Francesco Cavazzini, Elisabetta Abruzzese, Alessandro Isidori, Emilia Scalzulli, Domenico D’Agostino, Santino Caserta, Antonella Nardo, Roberto Massimo Lemoli, Daniela Cilloni, Monica Bocchia, Fabrizio Pane, Florian H. Heidel, Giuseppe A. Palumbo, Massimo Breccia, Elena M. Elli, Massimiliano Bonifacio

**Affiliations:** 1 IRCCS Azienda Ospedaliero-Universitaria di Bologna, Istituto di Ematologia “Seràgnoli”, Bologna, Italy; 2 Dipartimento di Medicina Specialistica, Diagnostica e Sperimentale, Università di Bologna, Bologna, Italy; 3 Hematology Unit, Department of Clinical and Molecular Sciences, DISCLIMO, Università Politecnica Delle Marche, Ancona, Italy; 4 University Hematology Division, Città della Salute e Della Scienza Hospital, Torino, Italy; 5 Division of Hematology and BMT, Department of Medicine, University of Udine, Udine, Italy; 6 Unit of Hematology, Department of Oncology, University of Torino, Torino, Italy; 7 Unit of Blood Diseases and Stem Cells Transplantation, Department of Clinical and Experimental Sciences, University of Brescia, ASST Spedali Civili of Brescia, Brescia, Italy; 8 Division of Hematology, Azienda Ospedaliera ’Bianchi Melacrino Morelli’, Reggio Calabria, Italy; 9 Ematologia, Ospedale Businco, Università Degli Studi di Cagliari, Cagliari, Italy; 10 Department of Clinical Medicine and Surgery, Federico II University Medical School, Naples, Italy; 11 Department of Hematology, Azienda USL - IRCCS di Reggio Emilia, Reggio Emilia, Italy; 12 Haematology and BMT Centre, Azienda Ospedaliero-Universitaria di Parma, Parma, Italy; 13 Unit of Hematology and Clinical Immunology, University of Padova, Padova, Italy; 14 Division of Hematology, University of Ferrara, Ferrara, Italy; 15 Division of Hematology, Ospedale S. Eugenio, Roma, Italy; 16 Hematology and Stem Cell Transplant Center, AORMN Hospital, Pesaro, Italy; 17 Hematology, Department of Translational and Precision Medicine, Az. Policlinico Umberto I-Sapienza University, Rome, Italy; 18 Department of Engineering for Innovation Medicine, Section of Innovation Biomedicine, Hematology Area, University of Verona, Verona, Italy; 19 Division of Hematology, Department of Human Pathology in Adulthood and Childhood “Gaetano Barresi”, University of Messina, Messina, Italy; 20 Department of Scienze Mediche, Chirurgiche e Tecnologie Avanzate “G.F. Ingrassia”, University of Catania, Catania, Italy; 21 IRCCS Ospedale Policlinico San Martino, Genoa, Italy; 22 Dipartimento di Medicina Interna e Specialità Mediche, Università di Genova, Genova, Italy; 23 Department of Clinical and Biological Sciences, University of Turin, Turin, Italy; 24 Hematology Unit, Azienda Ospedaliera Universitaria Senese, University of Siena, Siena, Italy; 25 Hematology, Hemostasis, Oncology and Stem Cell Transplantation, Hannover Medical School (MHH), Hannover, Germany; 26 Divisione di Ematologia e Unità Trapianto di Midollo, Fondazione IRCCS San Gerardo dei Tintori, Monza, Italy; 27 Institute of Hematology “L. and A. Seràgnoli”, IRCCS Azienda Ospedaliero-Universitaria di Bologna, Via Massarenti 9, Bologna (BO), 40138 Italy

**Keywords:** Myelofibrosis, Ruxolitinib, *CALR* mutation, Myeloproliferative neoplasms, *JAK2* mutation

## Abstract

Calreticulin (*CALR*) mutations are detected in around 20% of patients with primary and post-essential thrombocythemia myelofibrosis (MF). Regardless of driver mutations, patients with splenomegaly and symptoms are generally treated with *JAK2*-inhibitors, most commonly ruxolitinib. Recently, new therapies specifically targeting the *CALR* mutant clone have entered clinical investigation. To collect information on efficacy and safety of ruxolitinib in *CALR*-mutated patients, we report a sub-analysis of the “RUX-MF” (NCT06516406) study, comprising 135 *CALR*-mutated and 786 *JAK2*-mutated ruxolitinib-treated patients. Compared to *JAK2*-mutated patients, *CALR*-mutated patients started ruxolitinib with a more severe disease (higher peripheral blast counts, lower hemoglobin levels and worse marrow fibrosis) and after a longer median time from diagnosis (2.6 versus 0.7 years, *p* < 0.001). At 6 months, spleen responses were numerically inferior in *CALR*-mutated patients, who also had significantly lower rates of symptom responses (56.1% versus 66.7%, *p* = 0.04).

**Supplementary Information:**

The online version contains supplementary material available at 10.1007/s00277-025-06204-5.

## Introduction

Myelofibrosis (MF) is a challenging hematological malignancy characterized by bone marrow fibrosis, cytopenias, splenomegaly, and debilitating symptoms, with an overall severely reduced life expectation [1]. MF may present as primary disease (PMF) or secondary to Essential Thrombocythemia (ET) or Polycythemia Vera (PV) (SMF) [2].

Along with additional subclonal myeloid mutations, driver mutations in 3 genes that activate the *JAK/STAT* signaling pathway can be detected: Janus kinase 2 (*JAK2*) in 60–70%, calreticulin (*CALR*) in around 20%, and myeloproliferative leukemia virus (*MPL*) in 5%; 8–10% of patients lack all 3 driver mutations and are called “triple negative” [3].

*CALR* mutations occur mainly in exon 9 of the *CALR* gene and are divided into two categories: type 1-like (more common in MF), characterized by a 52 bp deletion and type 2-like (more common in ET), characterized by a 5 bp insertion [4]. In addition, there are a number of “atypical” mutations that do not belong to any of the two previous categories [5].

*CALR*-mutated PMF patients have distinct clinical features and outcomes compared to *JAK2*-mutated patients, presenting with younger age, lower levels of hemoglobin and white blood cells, higher platelet count and better survival rates [6]. Compared to type 2-like, type 1-like *CALR* mutations are associated with an improved survival in PMF [4, 7].

Ruxolitinib is a type-1 *JAK1/JAK2* inhibitor that has been used for the therapy of MF-related splenomegaly and symptoms for nearly 15 years. Ruxolitinib is not selective for the *JAK2V617F* mutation and has shown efficacy in MF patients regardless of the driver mutation [8, 9]. However, *JAK2V617F* variant allele frequency remains unchanged in many patients, and efficacy wanes over time, with only half of the responding patients still in response after 3 years [10, 11]. Also, *JAK2* inhibition causes on-target hematological toxicity, that may significantly limit the ruxolitinib dose and use [12].

All MF-associated *CALR* mutations result in a + 1 bp frameshift, leading to the translation of an altered C-terminus of the calreticulin protein. The novel C-terminus lacks the endoplasmic reticulum retention signal (KDEL domain) and consists predominantly of positively charged amino acids, resulting in ligand-independent activation of *MPL* and downstream *JAK–STAT* signaling pathway activation [13, 14].

Recent research has focused on immunotherapeutic approaches that specifically target the mutant calreticulin neoepitope while preserving normal hematopoiesis. INCA033989 is a novel human monoclonal class G immunoglobulin that was found to inhibit the mutant calreticulin-induced *MPL* signaling in murine Ba/F3 cells, with no effect on non-mutant cells. This agent is currently in early clinical development in *CALR*-mutated MF and ET patients (NCT06034002) [15].

The advent of new therapies specifically targeting the *CALR* mutant clone has urged the need to collect detailed information on therapies that are currently used in *CALR*-positive patients.

To provide critical insight into the comparative efficacy of ruxolitinib in *CALR*- and *JAK2*-mutated patients, we report the outcomes of 135 *CALR*-positive patients who received ruxolitinib in a real-world setting.

## Methods

### Patients’ collection and study design

After institutional review board approval, the “RUX-MF” retrospective study collected 1055 MF patients who received ruxolitinib outside clinical trials in 25 hematology centers that are dedicated to the treatment of myelofibrosis. All patients were in chronic phase at ruxolitinib start. Two separate analyses were performed: (1) including the total cohort of 921 patients with the *JAK2V167F* or *CALR* mutations; (2) including only patients transplant-eligible (age < 70 years at ruxolitinib start). This sub-analysis was performed considering that: (1) younger age of *CALR*-mutated patients compared to *JAK2*-mutated patients may influence survival outcomes; (2) younger patients are eligible for allogeneic stem cell transplantation (ASCT), which may significantly impact the treatment algorithm.

The list of the participating centers is available in **Supporting Information S1: Appendix**. All centers were asked to report, in an electronic case report form, their consecutive MF patients who received ruxolitinib according to standard clinical practice. The total number of medical files was reported by each center by data input into an electronic database developed to record all study data after the de-identification of the patients with an alphanumeric code to protect personal privacy. Data collected included patient demographics, comorbidities, medications, clinical-laboratory tests at diagnosis and during follow-up, date of ruxolitinib start and stop, type of MF therapies before and after RUX, duration of ruxolitinib treatment, and adverse events during the treatment. Any treatment decision, including starting ruxolitinib doses and dose adjustments over time, was at the physician’s discretion, based on patients’ characteristics and independent from participation to this study. After the first data entry, the follow-up information was validated with revision of clinical data, and specific queries were addressed to the participating center in case of inconsistent data.

All patients were followed from 2013 until death or to data cutoff (February 2024).

### Definitions

Diagnoses of PMF and SMF were made according to 2016 World Health Organization criteria (WHO) and International Working Group on Myelofibrosis Research and Treatment (IWG-MRT) criteria, respectively [16, 17]. 

The risk category was assessed at the time patients started on ruxolitinib according to the Dynamic International Prognostic Score System (DIPSS) for PMF [18] and Myelofibrosis Secondary to PV and ET-Prognostic Model (MYSEC-PM) [19]. Histologic examination was performed at local institutions; fibrosis was graded according to the European Consensus Grading System [20]. Unfavorable karyotype was categorized as previously described [21]. High molecular risk (HMR) pathogenetic mutations were defined as those including *ASXL1*, *SRSF2*, *EZH2*, *IDH1* and *IDH2*, *U2AF1* [22]. Anemia was defined according to Common Terminology Criteria for Adverse Events (CTCAE) [23].

A cytopenic phenotype was defined according to previously reported as at least one among Hb < 10 g/dL, platelet count < 100 × 10^9^/L or leukocytes count < 4 × 10^9^/L [24].

Leukemic transformation was defined by blast cells being at least 20% in peripheral blood and/or bone marrow according to WHO criteria [16]. MF-related symptoms were assessed using the 10-item Myeloproliferative Neoplasm Symptom Assessment Form Total Symptom Score (MPN10-TSS) [25]. Spleen and symptoms responses were routinely assessed by palpation and by periodical TSS evaluation, according to 2013 IWG-MRT criteria [17].

### Ethical aspects

The “RUX-MF” study (NCT06516406) was performed in accordance with the guidelines of the institutional review boards of the participating centers and the standards of the Helsinki Declaration. The promoter of this study was the IRCCS Azienda Ospedaliero-Universitaria S. Orsola-Malpighi, Bologna, which obtained approval from the Area Vasta Emilia Centro Ethics Committee (approval file number: 048/2022/Oss/AOUBo). The study was approved by the local ethics committee of participating centers (protocol code: RUX-MF) and has no commercial support.

### Statistical analysis

Continuous variables are expressed as medians and ranges or means and standard deviations, whereas categorical variables are presented as frequencies and percentages. We used the Wilcoxon-Mann-Whitney rank-sum test or the t-test for comparisons between groups, and associations between categorical variables (2-way tables) were tested using the Fisher exact test or the chi-square test, as appropriate. Continuous and categorical variables at MF diagnosis and at ruxolitinib start were compared using the Wilcoxon signed-rank test and the McNemar test, respectively.

A Poisson regression model was applied to calculate the incidence rate ratio (IRR) of leukemic transformation, ruxolitinib discontinuation and allogenic stem cell transplantation, together with 95% confidence interval (CI). The IRR was described as the number of events per 100 patient-years (%p-y).

Prognostic factors for spleen and symptoms response were evaluated using logistic regression model, while for survival were identified using univariate and multivariable Cox proportional hazards model. Multivariate logistic regression and Cox analysis were conducted on variables with P values < 0.05 at univariate analysis, to assess odds (OR) and hazard ratio (HR). To avoid the issue of multicollinearity and to remove highly correlated predictors from the model, collinearity among variables was detected using the Pearson correlation test. Variables that were associated with other factors in univariate analysis were excluded from the multivariable analysis. The following variables at ruxolitinib start were assessed: male sex, PMF (versus SMF), ruxolitinib dose < 15 bis in die (BID), lower than prescribing ruxolitinib dose according to platelet count, grade of marrow fibrosis ≥ 2, platelet count < 100 × 10^9^/L, leucocytes count > 25 × 10^9^/L and < 4 × 10^9^/L, hemoglobin levels < 10 g/dL, cytopenia, peripheral blasts count ≥ 1%, spleen length > 10 cm and TSS ≥ 20. In addition, symptoms and spleen response at 6 months were tested for overall survival.

Survival analyses comparing *CALR*- and *JAK2*-mutated patients were performed using Kaplan-Meier curves adjusted for delayed entry, and differences were evaluated using the log-rank test. Overall survival (OS) was calculated from the date of ruxolitinib start, to either death, last contact or ASCT. Event-free survival (EFS) was calculated from the date of ruxolitinib start to either death, leukemic transformation, ruxolitinib discontinuation, or last contact.

Tests were 2-sided, and P values < 0.05 were considered significant. Analyses were performed using STATA/SE software version 18.0 (StataCorp).

## Results

### Clinical and laboratory features at diagnosis and at ruxolitinib start according to *JAK2* or *CALR* mutation

Overall, 786 (74.5%) patients were *JAK2*-mutated and 135 (12.8%) had a *CALR* mutation. In the 78 evaluable patients, the *CALR* mutation was type 1-like in 66.7% (n. 52) and type 2-like in 30.8% (n. 24) of the population, while 2 patients had a novel-type *CALR* mutation (Supplemental Fig. 1).

At ruxolitinib start, compared to *JAK2*-mutated, *CALR*-mutated patients were younger (*p* = 0.02), had more frequently a marrow fibrosis grade 2–3 (*p* = 0.007), higher prevalence of HMR mutations (*p* = 0.04), higher percentages of peripheral blasts (*p* < 0.001) and lower median hemoglobin levels (*p* < 0.001). Conversely, the incidence of hyperleukocytosis (> 25 × 10^9^/L) was significantly lower in *CALR*-mutated patients (8.9% versus 16.5%, *p* = 0.02); median platelet counts, TSS scores and spleen lengths were comparable. Overall, 53.3% of *CALR*-mutated and 41.4% *JAK2*-mutated patients had a cytopenic phenotype (*p* = 0.009). Ruxolitinib starting doses were comparable, with 38.5% and 41.5% of patients starting with underdosed ruxolitinib in the two groups, respectively.

The median time from MF diagnosis to the start of ruxolitinib was significantly longer for *CALR*-mutated patients (2.6 years, range 0-32.9) compared to *JAK2*-mutated patients (0.7 years, range 0-28.1) (*p* < 0.001). Excluding post-PV patients, this difference remained statistically significant (0.9, range 0-28.1, *p* < 0.001). Between the time of diagnosis and the time of ruxolitinib start, clinical and hematological features worsened in both *JAK2* and *CALR* cohorts, with progressive increase of leukocytes, blast counts, splenomegaly and symptoms, and decrease of hemoglobin levels and platelet counts (Supplemental Table 1). However, *CALR*-mutated cohort had a higher incidence of patients with an increase of leucocytes (58.4% versus 46.0%, *JAK2*-mutated) and peripheral blasts count (35.9% versus 20.8%) and spleen length (52.5% versus 44.5%).

### Response to ruxolitinib and outcome according to *JAK2* or *CALR* mutation

At 6 months, there were no significant differences in spleen responses (*CALR*: 21.4% versus *JAK2*: 25.7%, *p* = 0.33), but symptoms response was significantly lower in *CALR*-mutated patients (56.1% versus 66.7%, *p* = 0.04) and overall anemia was higher (60.3% versus 50.3%, *p* = 0.04). Treatment-emergent anemia (*CALR*: 35.7% versus *JAK2*: 30.4%, *p* = 0.37), overall and treatment-emergent thrombocytopenia were comparable in the two groups (*p* = 0.49 and *p* = 0.60, respectively).

During a median follow-up from ruxolitinib start of 3.4 years (range: 0.1–15.1), 597 patients discontinued ruxolitinib, 464 (50.4%) died, 130 (14.1%) had a leukemic transformation and 91 (9.9%) underwent ASCT.

Causes of ruxolitinib discontinuation were comparable in the two groups (intolerance: 48.2% overall, *JAK2*: 48.3% versus *CALR*: 47.7%; Resistance 90.7% overall, *JAK2*: 90.3% versus *CALR*: 93.2%) with comparable percentages of patients with both intolerance and resistance (42.4% overall; *CALR*: 43.2% versus *JAK2*: 42.3%). However, a cytopenic phenotype was significantly more frequent in *CALR*-mutated patients (85.4%) than in *JAK2*-mutated patients (75.0%, *p* = 0.03).

Incidence rates of ASCT was significantly higher in *CALR*-mutated patients (5.8 versus JAK2: 2.1%p-y, *p* < 0.001), while other events had comparable frequency.

In the 135 patients with the *CALR* mutation, no factors were found to be associated with spleen or symptom response, including larger splenomegaly and delayed ruxolitinib start. However, hemoglobin below 10 g/dL and a high symptom burden (TSS ≥ 20) significantly correlated with worse survival (Supplemental Table 2).

The survival analysis was repeated including only the 72 *CALR*-mutated patients who started ruxolitinib therapy more than two years after diagnosis. In this subgroup, anemia (HR: 1.92, 95% CI: 1.02–3.79, p-value: 0.05) and the use of a reduced ruxolitinib starting dose (HR: 2.29, 95% CI: 1.15–4.56, p-value: 0.02) were associated with poorer overall survival in multivariate analysis.

### Characteristics and treatment outcomes in transplant-eligible and older-age patients

Overall, 523 (56.8%) patients started ruxolitinib while younger than 70 years (transplant-age cohort) and 398 (43.2%) received ruxolitinib at an older, no transplant-permissive age (older-age cohort).

Patients’ characteristics according to age at ruxolitinib start are summarized in Table 1.

**Table 1:** Baseline characteristics of older-age and transplant-age *CALR* versus *JAK2*-mutated patients

	Overall Cohort			Transplant-age Cohort			Older-age Cohort		
	CALR(*n*.135)	JAK2(*n*. 786)	p	CALR(*n*. 82)	JAK2(*n*. 441)	p	CALR(*n*. 53)	JAK2(*n*. 345)	p
**Median age, years (range)**	67.5 (24.0-87.2)	68.5 (26.5–92.6)	**0.02**	60.6 (24.0-69.8)	62.8 (26.5–69.9)	0.17	74.1 (70.0-87.2)	75.8 (70.0-92.6)	**0.005**
**Male Sex, n. (%)**	77 (57.0%)	445 (56.6%)	0.93	51 (62.2%)	243 (55.1%)	0.24	26 (49.1%)	202 (58.4%)	0.20
**PMF, n. (%)**	79 (58.5%)	387 (49.2%)	0.06	45 (54.9%)	218 (49.4%)	0.38	34 (64.2%)	169 (49.0%)	**0.04**
Post-PV MF, n. (%)	0	256 (32.6%)		0	145 (32.9%)		0	111 (32.2%)	
Post-ET MF, n. (%)	56 (41.5%)	143 (18.2%)		37 (45.1%)		78 (17.7%)	19 (35.8%)	65 (18.8%)	
RUX starting daily dose, n. (%), *on*									
10–20 mg	53 (39.3%)	307 (39.1%)	0.93	30 (36.6%)	158 (35.8%)	0.90	23 (43.4%)	150 (43.5%)	0.93
30–40 mg	82 (60.7%)	479 (60.9%)		52 (63.4%)	283 (64.2%)		30 (56.6%)	479 (56.5%)	
**Lower than prescribing dose, n. (%)**	52 (38.5%)	326 (41.5%)	0.52	27 (32.9%)	172 (39.2%)	0.28	25 (47.2%)	154 (44.6%)	0.67
Dose reduction at 6 months, n. (%)	24/75 (32.0%)	144/444 (32.4%)	0.94	14/46 (30.4%)	77/273 (28.2%)	0.76	10/29 (34.5%)	67/171 (39.2%)	0.63
**Grade of marrow fibrosis < 2, n. (%)**	17/127 (13.4%)	180/746 (24.1%)	0.007	12/77 (15.6%)	113/417 (27.1%)	0.03	5/50 (10.0%)	68/330 (20.6%)	0.07
**DIPSS/MYSEC–PM score, n. (%)**									
Intermediate–1	72 (53.3%)	464 (59.0%)	0.29	52 (63.4%)	301 (68.3%)	0.73	20 (37.7%)	164 (47.5%)	0.17
Intermediate–2	47 (34.8%)	257 (32.7%)		25 (30.5%)	117 (26.6%)		22 (41.5%)	140 (40.6%)	
High	16 (11.9%)	65 (8.3%)		5 (6.1%)	23 (5.2%)		11 (20.8%)	41 (11.9%)	
**HMR mutation, n. (%)**	32/51 (62.8%)	94/201 (46.8%)	0.04	24/37 (64.9%)	63/135 (46.7%)	0.05	8/14 (57.1%)	31/66 (47.0%)	0.49
**Platelet count, median (range), x 109/L**	310 (53– 1887)	261 (14– 1632)	0.18	250.5 (53–982)	264.5 (14–1425)	0.64	359 (53– 1887)	252 (26– 1632)	0.01
Platelet count < 100 x 109/L	15 (11.1%)	74 (9.4%)	0.55	11 (13.4%)	37 (8.4%)	0.15	4 (7.6%)	37 (10.7%)	0.55
**Leukocytes, median (range), x 109/L,**	8.46 (2.1– 71.1)	12 (1.1– 155)	**<0.001**	8.7 (2.9–71.1)	11 (2.1–80)	**0.01**	8.3 (2.1– 42.9)	13.5 (1.1– 155)	**<0.001**
Leukocytes >25 x 109/L, n. (%)	12 (8.9%)	130 (16.5%)	**0.02**	6 (7.3%)	64 (14.5%)	0.08	6 (11.3%)	66 (19.1%)	0.17
Leukocytes <4 x 109/L, n. (%)	13 (9.6%)	63 (8.0%)	0.54	8 (9.8%)	38 (8.6%)	0.76	5 (9.4%)	25 (7.2%)	0.54
**Hemoglobin, median (range), g/dL**	10.4 (5.6– 14.5)	11.2 (5.7– 18.3)	**<0.001**	10.6 (5.6–14.4)	11.5 (6.0–18.3)	**0.001**	10.0 (6.6– 14.5)	10.6 (5.7– 16.7)	0.12
Hemoglobin < 10 g/dL, n. (%)	59 (43.7%)	263 (33.5%)	**0.02**	33 (40.2%)	129 (29.3%)	**0.05**	26 (49.1%)	134 (38.8%)	0.16
**Cytopenic Phenotype, n. (%)**	72 (53.3%)	325 (41.4%)	**0.009**	42 (51.2%)	158 (35.8%)	**0.009**	30 (56.6%)	167 (48.4%)	0.26
**Blasts, mean ± SD, %**	1.6 ± 2.0	0.9 ± 1.6	**<0.001**	1.6 ± 2.1	0.9 ± 1.6	**<0.001**	28 (52.8%)	117 (33.9%)	**0.005**
**(range), cm**									
**Blasts, mean ± SD, %**	8 (0– 30)	10 (0– 5)	0.31	9 (0–30)	10 (0–35)	0.79	8 (0– 25)	10 (0– 35)	0.22
Spleen > 10 cm, n. (%)	57 (42.2%)	353 (44.9%)	0.54	36/81 (44.4%)	188/436 (43.1%)	0.83	21 (39.6%)	165 (47.8%)	0.24
**Total Symptoms Score, median (range)**	20 (0– 87)	20 (0– 100)	0.64	20 (0–87)	20 (0–100)	0.10	24 (0– 80)	20 (0– 100)	0.16
Total Symptoms Score ≥ 20, n. (%)	74/123 (60.2%)	457/741 (61.7%)	0.75	40/76 (52.6%)	258/416 (62.0%)	0.12	34/47 (72.3%)	199/325 (61.2%)	0.14
**Time between MF diagnosis and RUX start,**									
**median (range), years**	2.6 (0– 32.9)	0.7 (0– 28.1)	**<0.001**	2.8 (0– 32.9)	0.6 (0– 28.1)	**<0.001**	2.0 (0.05– 22.3)	0.9 (0– 23.5)	**0.02**
>2 years, n. (%)	72 (53.3%)	271 (34.5%)	**<0.001**	46 (56.1%)	150 (34.0%)	**<0.001**	26 (49.1%)	121 (35.1%)	**0.05**

PMF, Primary Myelofibrosis; PV: Polycythemia Vera; ET, Essential Thrombocythemia; RUX, ruxolitinib; DIPSS, Dynamic International Prognostic Scoring System; MYSEC-PM, Myelofibrosis Secondary to PV and ET-Prognostic Model; HMR, High Molecular Risk

In the transplant–age cohort, a baseline higher grade of marrow fibrosis (*p* = 0.03), higher rate of HMR mutations (*p* = 0.05), higher peripheral blast percentage (*p* < 0.001) was noted in *CALR*-mutated patients. Conversely, they had lower leucocytes count (*p* = 0.01) and hemoglobin levels (*p* = 0.001) and longer time from MF diagnosis to the start of ruxolitinib (*p* < 0.001). Overall, a cytopenic phenotype was observed in 51.2% and 35.9% of *CALR*- and *JAK2*-mutated patients, respectively (*p* = 0.009).

In the older-age cohort, median age and leukocyte count at ruxolitinib start were significantly higher in *JAK2*-mutated patients (*p* = 0.005), whereas median platelet and blast count were higher in *CALR*-mutated patients (*p* = 0.01 and *p* = 0.006).

Comparison of patient characteristics between the transplant-age and older-age cohorts is summarized in Supplemental Table 3.

Response rates and survival outcomes in transplant-age and older-age groups are detailed in Table 2.

**Table 2:** Outcomes in older-age and transplant-age *CALR* versus *JAK2*-mutated patients

	Overall Cohort			Transplant-age Cohort			Older-age Cohort		
	CALR(*n*.135)	JAK2(*n*. 786)	**p**	CALR(*n*. 82)	JAK2(*n*. 441)	**p**	CALR(*n*. 53)	JAK2(*n*. 345)	**p**
Spleen response at 6 months, n. (%)	24/112 (21.4%)	170/661 (25.7%)	0.33	16/68 (23.5%)	91/376 (24.2%)	0.91 0.08	8/44 (18.2%)	79/283 (27.9%)	0.17
Spleen response at any time, n. (%)	45/118 (38.1%)	328/702 (46.7%)	0.08	26/71 (36.6%)	192/400 (48.0%)	0.08	19/47 (40.4%)	136/302 (45.0%)	0.08
Symptoms response at 6 months, n. (%)	55/98 (56.1%)	398/597 (66.7%)	**0.04**	31/59 (52.5%)	230/332 (69.3%)	**0.01**	24/39 (61.5%)	168/265 (63.4%)	0.82
Any grade hematological toxicity at 6 months									
Overall anemia, n. (%)	73/121 (60.3%)	348/692 (50.3%)	**0.04**	41/74 (55.4%)	169/394 (42.9%)	**0.05**	32/47 (68.1%)	176/298 (59.1%)	0.24
Treatment-emergent anemia, n. (%)	25/70 (35.7%)	141/464 (30.4%)	0.37	15/45 (33.3%)	67/277 (24.2%)	0.19	10/25 (40.0%)	74/187 (39.6%)	0.97
Overall thrombocytopenia, n. (%)	25/120 (20.8%)	164/691 (23.7%)	0.49	16/73 (21.9%)	77/393 (19.6%)	0.65	9/47 (19.2%)	87/298 (29.2%)	0.15
Treatment-emergent thrombocytopenia, n. (%)	18/112 (16.1%)	116/640 (18.1%)	0.60	12/67 (17.9%)	56/369 (15.2%)	0.57	6/45 (13.3%)	60/271 (22.1%)	0.18
**Allogeneic stem cell transplantation, n. (%)**	26 (19.3%)	65 (8.3%)	**< 0.001**	26 (31.7%)	65 (14.7%)	**< 0.001**	0	0	/
IRR (%p-y)	5.8	2.1	**< 0.001**	9.2	3.4	**< 0.001**	0	0	/
**Leukemic transformation, n. (%)** *IRR (%p-y)*	16 (11.9%) 3.2	114 (14.5%) 3.6	0.41 0.62	7 (8.5%) 2.0	67 (15.2%) 3.3	0.11 0.21	9 (17.0%) 5.6	47 (13.6%) 4.2	0.51 0.43
**Leukemic transformation, n. (%)**	16 (11.9%)	114 (14.5%)	0.41	7 (8.5%)	67 (15.2%)	0.11	9 (17.0%)	47 (13.6%)	0.51
IRR (%p-y)	24.0	20.0	0.11	24.0	16.1	**0.009**	24.0	26.3	0.62
Events, n. (%)	96 (71.1%)	523 (66.5%)	0.296	58 (70.7%)	266 (60.3%)	0.08	38 (71.7%)	257 (74.5%)	0.69
IRR (%p-y)	25.3	20.7	0.07	24.9	16.9	**0.01**	26.0	27.1	0.83
Death, n. (%)	68 (50.4%)	396 (50.4%)	0.99	36 (43.9%)	158 (35.8%)	0.17	31 (58.5%)	238 (69.0%)	0.14
IRR (%p-y)	13.1	12.3	0.59	10.6	7.6	0.07	18.4	20.9	0.52

At 6 months, spleen responses were comparable in *CALR* and *JAK2*-mutated patients both in the transplant-age (23.5% versus 24.2%, *p* = 0.91) and in the older-age groups (18.2% versus 27.9%, *p* = 0.17). However, in transplant-age patients with the *CALR* mutation, symptoms response was significantly lower (52.5% versus 69.3% in *JAK2*-mutated patients, *p* = 0.01) and overall anemia rate was higher (55.4% versus 42.9%, *p* = 0.05).

### Survival outcomes in transplant-eligible patients

In the 523 transplant-eligible patients, the IRR of leukemic transformation was comparable between *CALR* and *JAK2*-mutated patients (2.0 versus 3.3%p-y, *p* = 0.21), while the rates of ruxolitinib discontinuation were higher in *CALR*-mutated patients (24.0 versus 16.1%p-y, *p* = 0.009). ASCT was performed in 91 patients (17.4%), with an IRR of 8.9 and 3.4%p-y in *CALR* and *JAK2*-mutated patients, respectively (*p* < 0.001).

**Fig. 1:**
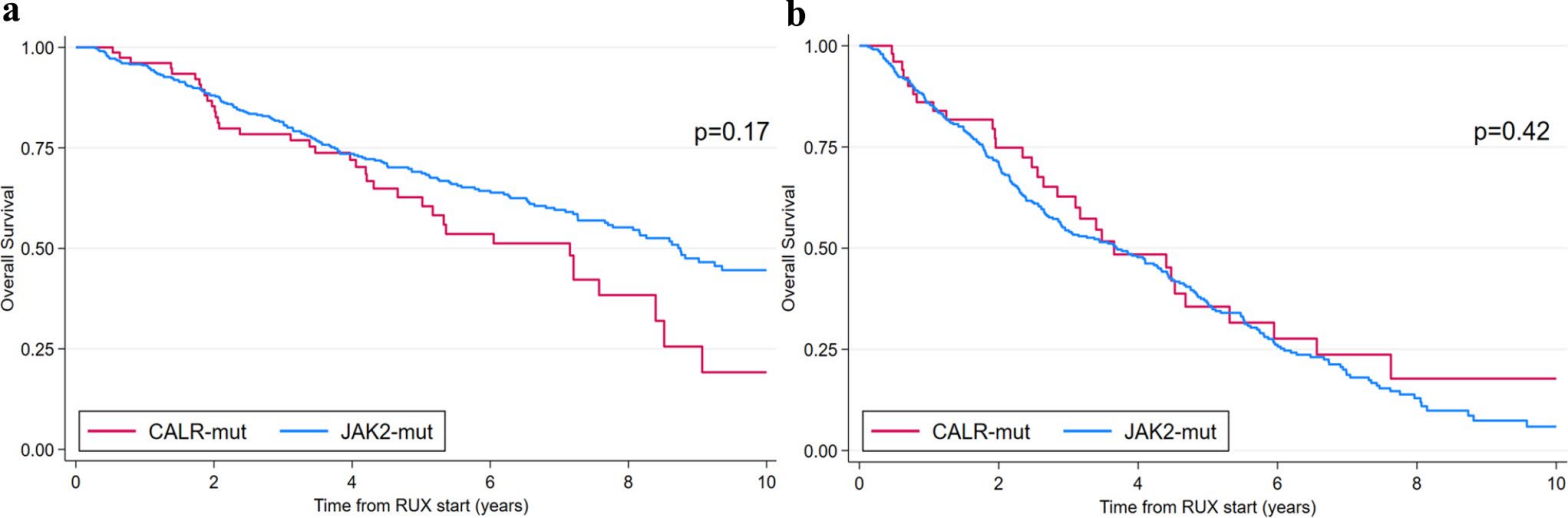
Overall Survival in comparison between *CALR*- and *JAK2*-mutated transplant-age patients (**a**) and older-age patients (**b**). RUX, ruxolitinib; mut, mutated

OS at 3 and 5 years was 79.7% and 63.7% for *CALR*-mutated patients and 81.5% and 69.1% for *JAK2*-mutated patients, respectively (log-rank test *p* = 0.17) (Fig. 1a). The median OS for *CALR*-mutated patients was 7.2 years (95% CI: 5.0–8.4), while for *JAK2*-mutated was 8.4 years (95% CI: 7.7–10.5).

### Survival outcomes in older-age patients

In the 398 older-age patients, the IRR of leukemic transformation (5.6 versus 4.2%p-y, *p* = 0.43) and of ruxolitinib discontinuation (24.0 versus 26.3%p-y, *p* = 0.62) were comparable between *CALR* and *JAK2*-mutated patients. None of the patients in either of the two groups underwent ASCT.

OS at 3 and 5 years was 62.8% and 35.5% for *CALR*-mutated patients and 54.4% and 36.2% for *JAK2*-mutated patients, respectively (log-rank test *p* = 0.07) (Fig. 1b).

In *CALR*-mutated patients, the median OS and EFS were 3.7 years (95% CI: 2.6–5.3) and 3.0 (95% CI: 2.0–3.8), comparably to *JAK2*-mutated patients (OS 3.7 years and EFS 2.3 years).

## Discussion

Our study highlights several critical aspects of *CALR*-mutated MF patients undergoing ruxolitinib therapy.

Firstly, *CALR*-mutated patients, regardless of age group, started therapy with the *JAK2* inhibitor with a more severe clinical burden characterized by more frequent anemia, higher blast percentages, and higher presence of high-risk biological features including increased marrow fibrosis and presence of HMR mutations.

While clinical and laboratory features worsened between diagnosis and ruxolitinib start in both *CALR* and *JAK2* patients, confirming the progressive nature of the disease, *CALR*-mutated patients had the greatest worsening, with a higher number of patients acquiring anemia and increasing the peripheral blast count. This was possibly due to the striking difference in the median time from diagnosis to ruxolitinib initiation: *CALR*-mutated patients waited significantly longer (median time: 2.6 years) compared to *JAK2*-mutated patients (0.7 years, *p* < 0.001). This prolonged time may certainly reflect a more indolent nature of *CALR*-mutated myelofibrosis, or perhaps an initial reluctance to use ruxolitinib in *JAK2*-negative patients.

Nonetheless, spleen and symptom responses were lower, and anemia was higher in *CALR*-mutated patients after 6 months of therapy. In the *CALR*-positive cohort, we could not detect any prognostic factors for spleen or symptom response, including larger splenomegaly and delayed ruxolitinib start. However, hemoglobin below 10 g/dL and a high symptom burden significantly correlated with worse survival. In the 72 *CALR*-positive patients with delayed (> 2 years) ruxolitinib start, anemia and a reduced ruxolitinib starting dose were associated with poorer overall survival. While it may be difficult to find prognostic factors for response in small cohorts, the identification of anemia as a major contributor to worse prognosis also in *CALR*-mutated patients confirms that anemia should be a key target of MF therapies. We also confirm that appropriate ruxolitinib dosing may have a beneficial effect on outcome [26, 27].

Additionally, the observation of a lower symptoms’ response in *CALR*-positive patients constitutes a novel finding that may be probably attributable to reduced hemoglobin levels, both prior to and during ruxolitinib therapy. This finding confirms that anemia is not only a significant predictor of reduced survival, but also of diminished quality of life and heightened severity of symptoms reported by patients [28–32].

The scope and potential findings of this retrospective study precludes determination of the most efficacious interventions for the management of anemia. Nevertheless, the combined use of danazol, erythropoiesis-stimulating agents, iron chelation therapy and ruxolitinib dose optimization have been proposed as a means of mitigating the burden of anemia and may be useful in *CALR*-mutated patients [33–37]. The use of alternative *JAK2* inhibitors, which have been shown to have reduced hematological toxicity or even the potential to benefit anemia, could also be considered [38–40].

Unlike previous observations in patients followed from diagnosis [3], we failed to confirm that *CALR*-mutated patients demonstrate a more prolonged survival during ruxolitinib therapy. In this setting, we have observed that *CALR*-mutated patients started ruxolitinib with a more severe disease burden and after a longer time from diagnosis compared to *JAK2*-mutated patients. The impact of these factors on treatment success and outcome is not yet fully elucidated, but they may potentially counterbalance the positive effect of the calreticulin mutation over the *JAK2* mutation. This observation potentially explains the observed overlap in survival curves between the two groups.

However, the rationale behind the decision to delay the initiation of ruxolitinib treatment in *CALR*-mutated patients, as opposed to *JAK2*-mutated patients, remains unclear. Nevertheless, the present report may serve to enhance medical education, thereby stimulating earlier treatment also in *CALR*-positive patients [27, 41].

In transplant-age patients, the presence of the *CALR* mutation was associated with significantly higher rates of ruxolitinib discontinuation, despite comparable degrees of splenomegaly/symptoms and reasons for ruxolitinib stop. However, in most cases, *CALR*-mutated patients displayed a cytopenic phenotype, that possibly prevented medical approaches with conventional or investigational therapy. Indeed, cytopenia significantly limits the available treatment options [40, 42]. As a result, it is plausible that cytopenia instead urged toward the transplant procedure.

Interestingly, the comparable rates of leukemic transformation between *CALR*- and *JAK2*-mutated patients indicate that the primary challenge in *CALR*-mutated patients lies in managing their higher baseline disease burden and maintaining long-term ruxolitinib efficacy. Therefore, early identification of high-risk features such as baseline anemia can help stratify patients and guide prompt treatment decisions.

Overall, despite the initial benefits of ruxolitinib, *CALR*-mutated patients may require more innovative therapeutic interventions to achieve optimal outcomes. This further emphasizes the necessity of exploring alternative or adjunctive therapies tailored specifically for *CALR*-mutated individuals [15].

This study has well-known limitations that are mainly related to its retrospective and non-randomized nature, with potentially inadequate recognition of unidentified additional parameters that could influence treatment and survival outcomes. Also, the relatively low number of *CALR*-mutated patients with complete molecular information (e.g., type and variant allele frequency, HMR mutations) prevented subgroup analyses that would add knowledge to this comparison. The lack of accurate molecular analysis also exposes a significant need for improving molecular evaluation of MPN patients in many Hematology Centers.

Importantly, the decision to initiate and to discontinue ruxolitinib, as well as the decision to perform the transplant, were not standardized or randomized, but were variably based on individual clinical judgement, patient preference or local practices. This variability limits the ability to draw firm conclusions about the impact of driver mutations in the treatment algorithm after ruxolitinib discontinuation.

## Conclusion

The current study provides real-world observations in *CALR*-mutated MF patients with splenomegaly and/or symptoms requiring therapy with *JAK2* inhibitors. These data may represent a valid source document for interpreting current literature and planning future studies.

## Electronic supplementary material

Below is the link to the electronic supplementary material.

Supplementary Material 1

## Data Availability

The data that support the findings of this study are available from the corresponding author upon reasonable request to the corresponding author (francesca.palandri@unibo.it). 10.5281/zenodo.13739551.
