# Supplementary material for: Impact of calreticulin mutations on treatment and survival outcomes in myelofibrosis during ruxolitinib therapy
Source: Ann Hematol. 2025 Jan 20;104(1):241–51. doi: 10.1007/s00277-025-06204-5 (PMC11868333; doi:10.1007/s00277-025-06204-5)
Supplement: Supplementary file 1 — Supplementary Material 1 [file 277_2025_6204_MOESM1_ESM.pdf]

Supporting Information S1: Appendix

Center	Contributors
**IRCCS Azienda Ospedaliero-Universitaria di Bologna, Bologna, Italy / Dipartimento di Medicina Specialistica, Diagnostica e Sperimentale, Università di Bologna, Bologna, Italy**	Francesca Palandri, Filippo Branzanti Alessandra Dedola, Pier Luigi Zinzani
**Hematology Division, Foundation IRCCS Ca' Granda Ospedale Maggiore Policlinico, Milan**	Alessandra Iurlo, Daniele Cattaneo
**Hematology, S. Eugenio Hospital, Tor Vergata University, ASL Roma2, Rome, Italy**	Elisabetta Abruzzese, Malgorzata M. Trawinska, Vanessa Velotta
**Division of Cellular Biotechnologies and Hematology, University Sapienza, Rome, Italy**	Massimo Breccia, Emilia Scalzulli Gioia Colafigli
**Division of Hematology, Città della Salute e della Scienza Hospital, Torino, Italy**	Giulia Benevolo, Francesca Pirillo
**Hematology Division and Bone marrow transplant unit, Fondazione IRCCS San Gerardo dei Tintori, Monza, Italy**	Elena M. Elli, Cavalca Fabrizio Veronica Guglielmana
**Division of Hematology, University of Ferrara, Italy**	Francesco Cavazzini, Antonio Cuneo
**Unit of Hematology and Clinical Immunology, University of Padova, Padova, Italy**	Gianni Binotto, Gianpietro Semenzato Fabio D'Amore, Antonio Maroccia
**Department of Hematology, Azienda USL—IRCCS di Reggio Emilia, Reggio Emilia, Italy**	Alessia Tieghi, Katia Codeluppi Domenico Penna, Elisabetta Lugli
**Division of Hematology and BMT, Azienda Sanitaria Universitaria Integrata di Udine, Italy**	Mario Tiribelli, Luca Tosoni
**Division of Hematology, AUSL di Piacenza, Piacenza, Italy**	Costanza Bosi, Nicole Codeluppi Roberta Michelotti
**Hematology Unit, Azienda Ospedaliera Universitaria Senese, University of Siena, Siena, Italy**	Monica Bocchia
**Department of Engineering for Innovation Medicine, Section of Innovation Biomedicine, Hematology Area, University of Verona**	Massimiliano Bonifacio, Mauro Krampera Luigi Scaffidi, Andrea Bernardelli Domenico D'Agostino, Alessia Moioli Eleonora Santoni, Alda Strazimiri
**Department of Clinical Medicine and Surgery, Federico II University Medical School, Naples, Italy**	Novella Pugliese Fabrizio Pane
**Ematologia, Ospedale Businco, Università degli studi di Cagliari, Cagliari, Italy**	Giovanni Caocci, Maria Pina Simula Olga Mulas, Alessandro Costa
**Division of Hematology, Azienda Ospedaliero-Universitaria di Parma, Parma, Italy**	Monica Crugnola Elena Masselli
**Hematology and Stem Cell Transplant Center, AORMN Hospital, Pesaro, Italy**	Alessandro Isidori
**Division of Hematology, Azienda Ospedaliera 'Bianchi Melacrino Morelli', Reggio Calabria, Italy**	Bruno Martino
**Unit of Blood Diseases and Stem Cell Transplantation, ASST Spedali Civili di Brescia, Brescia, Italy**	Mirko Farina, Domenico Russo Lisa Gandolfi
**IRCCS Policlinico San Martino, Genova, Italy/Clinic of Hematology, Department of Internal Medicine (DiMI), Genova, Italy**	Roberto M. Lemoli, Maurizio Miglino Maria Grazia Ciardo
**Department of Scienze Mediche, Chirurgiche e Tecnologie Avanzate "G.F. Ingrassia", University of Catania, Italy**	Giuseppe A. Palumbo
**Postgraduate School of Hematology, University of Catania**	Andrea Duminuco
**Haematology Division, Department of Clinical and Biological Sciences, Ospedale San Luigi di Orbassano, University of Turin, Orbassano, Italy**	Daniela Cilloni
**Division of Hematology, Onco-hematologic Department, AUSL della Romagna, Ravenna**	Alessandra D'Addio
**Hematology Unit, Department of Clinical and Molecular Sciences, DISCLIMO, Università Politecnica delle Marche, Ancona, Italy**	Erika Morsia

Supplemental Table 1: Clinical and Laboratory Characteristics at Diagnosis and Start of Ruxolitinib Treatment in *CALR*- and *JAK2*-mutated patients in transplant-age

	Characteristics	At Diagnosis	At ruxolitinib start	With Increased Continuous Values or With Shift of a Dichotomous Value From “No” to “Yes”	With Decreased Continuous Values or With Shift of a Dichotomous Value From “Yes” to “No”	With Stable Values	p-value
***CALR* mutated patients**	Hemoglobin, median (range), g/dL	11.1 (7.4 – 14.6)	10.6 (5.6 – 14.4)	25 (32.1%)	43 (55.1%)	10 (12.8%)	**0.002**
	Hemoglobin <10 g/dL, n. (%)	17/78 (21.8%)	33 (40.2%)	16 (20.5%)	2 (2.6%)	60 (76.9%)	**0.001**
	Leucocytes, median (range), $\times 10^9$/L	8.7 (3 – 41.8)	8.7 (2.9 – 71.1)	45 (58.4%)	26 (33.8%)	6 (7.8%)	**0.03**
	Leucocytes > 25$\times 10^9$/L, n. (%)	1/77 (1.3%)	8 (9.8%)	5 (6.5%)	0	72 (93.5%)	0.06
	Blasts, mean (range), %	0.9 (0 – 9)	1.6 (0 – 9)	28 (35.9%)	10 (12.8%)	40 (51.3%)	**0.002**
	Blasts ≥ 1%, n. (%)	31/79 (39.2%)	45/81 (55.6%)	19 (24.4%)	6 (7.7%)	63 (80.8%)	**0.01**
	Platelets, median (range), $\times 10^9$/L	362 (46 – 1268)	250.5 (53 – 982)	15 (19.5%)	54 (70.1%)	8 (10.4%)	**<0.001**
	Platelets < 100$\times 10^9$/L, n. (%)	5/77 (6.5%)	11 (13.4%)	6 (7.8%)	0	71 (92.2%)	**0.03**
	Spleen, median (range), cm BCM	6 (0 – 30)	9 (0 – 30)	42 (52.5%)	9 (11.2%)	29 (36.3%)	**<0.001**
	Spleen > 10 cm BCM, n. (%)	22/81 (27.2%)	36/81 (44.4%)	14 (17.5%)	0	66 (82.5%)	**<0.001**
***JAK2* mutated patients**	Hemoglobin, median (range), g/dL	12.1 (5.0-19.5)	11.5 (6.0-18.3)	150 (35.1%)	237 (55.5%)	40 (9.4%)	**<0.001**
	Hb < 10 g/dL, n. (%)	92/430 (21.4%)	129 (29.3%)	58 (13.6%)	23 (5.4%)	346 (81.0%)	**<0.001**
	Leucocytes, median (range), $\times 10^9$/L	10.5 (2.7 – 97.6)	11 (2.1 – 80)	196 (46.0%)	201 (47.2%)	29 (6.8%)	0.99
	Leucocytes > 25$\times 10^9$/L, n. (%)	50/429 (11.7%)	38 (8.6%)	35 (8.2%)	20 (4.7%)	371 (87.1%)	0.06
	Blasts, mean (range), %	0.7 (0 – 10)	0.9 (0 – 10)	86 (20.8%)	45 (10.9%)	283 (68.4%)	**<0.001**
	Blasts ≥ 1%, n. (%)	118/426 (27.7%)	147/425 (34.6%)	60 (14.5%)	30 (7.2%)	324 (78.3%)	**0.002**
	Platelets, median (range), $\times 10^9$/L	328 (42 – 1521)	264.5 (14 – 1425)	155 (36.2%)	236 (55.1%)	37 (8.6%)	**<0.001**
	Platelets < 100$\times 10^9$/L, n. (%)	19/431 (4.4%)	37 (8.4%)	26 (6.1%)	8 (1.9%)	394 (92.0%)	**0.003**
	Spleen, median (range), cm BCM	8 (0 – 33)	10 (0 – 35)	191 (44.5%)	59 (13.8%)	179 (41.7%)	**<0.001**
	Spleen > 10 cm BCM, n. (%)	148/441 (34.2%)	188/436 (43.1%)	57 (13.3%)	17 (4.0%)	355 (82.7%)	**<0.001**

Supplemental Table 1: BCM, Below Costal Margin

Supplemental Figure 1: Patients' disposition

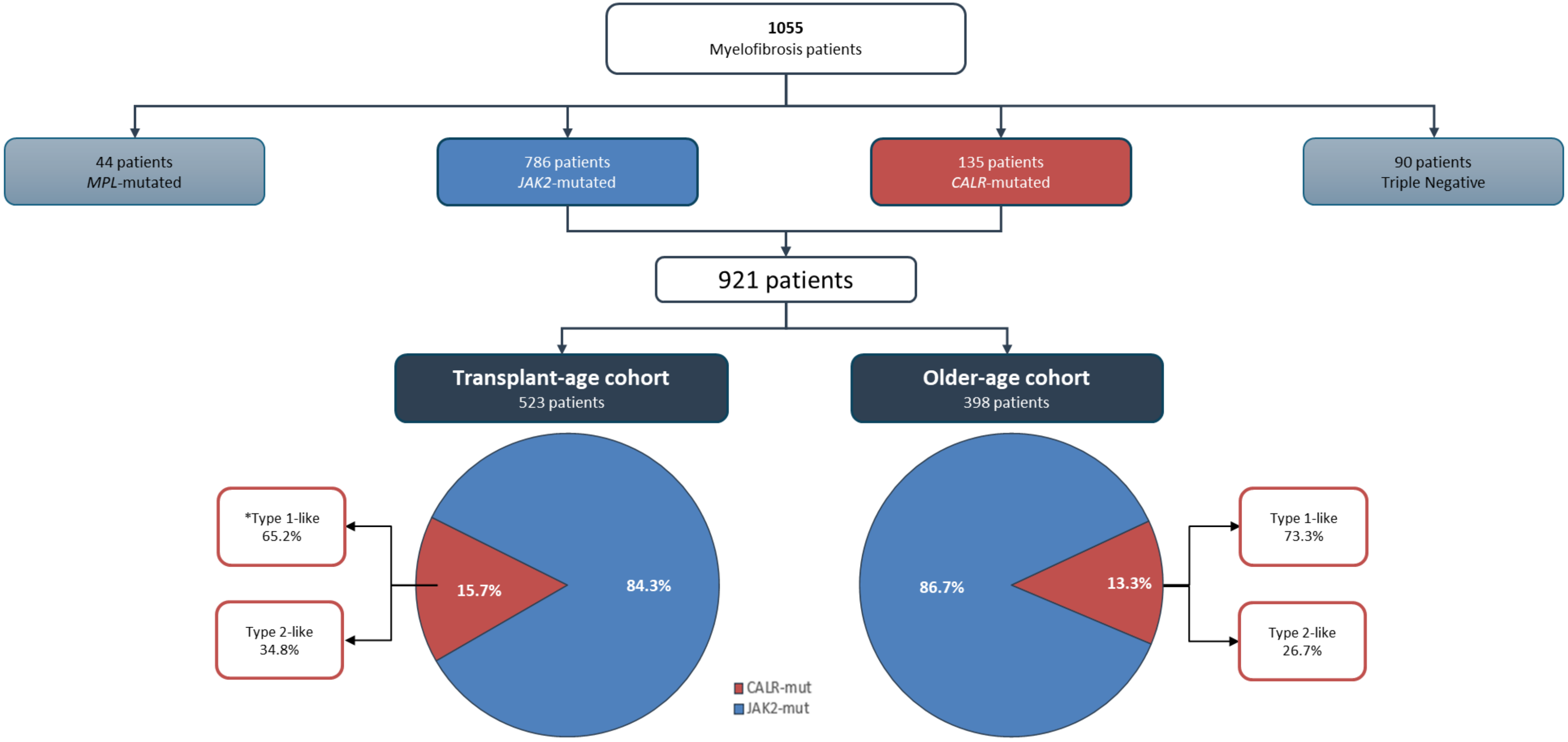

*CALR-type evaluation is available only for 76/135 patients (56.3%) (46 transplant-age; 30 older-age)

Supplemental Table 2: Prognostic Factors Associated with Response and Survival in *CALR*-Mutated Patients

***CALR*-mutated patients (n. 135)**	***Association with Spleen Response***	***Association with Symptoms Response***	***Association with Overall Survival***	
**Variables (at ruxolitinib start)**	**Univariate (OR, 95% CI, p-value)**	**Univariate (OR, 95% CI, p-value)**	**Univariate (HR, 95% CI, p-value)**	**Multivariate (HR, 95% CI, p-value)**
Male sex (vs Female sex)	0.57, 0.23 – 1.42, 0.23	1.79, 0.79 – 4.07, 0.17	0.96, 0.59 – 1.55, 0.86	
Primary vs Secondary myelofibrosis	1.32, 0.51 – 3.42, 0.57	1.01, 0.45 – 2.25, 0.99	0.91, 0.57 – 1.47, 0.71	
Ruxolitinib dose <15 BID	0.92, 0.35 – 2.39, 0.86	0.75, 0.33 – 1.75, 0.51	1.26, 0.76 – 2.10, 0.38	
Lower than prescribing ruxolitinib starting dose	0.83, 0.32 – 2.16, 0.71	0.57, 0.25 – 1.32, 0.19	1.37, 0.82 – 2.29, 0.24	
Platelet count <100 x10^9/L	0.91, 0.18 – 4.59, 0.90	1.19, 0.31 – 4.53, 0.79	1.28, 0.63 – 2.58, 0.49	
Leukocytes count >25 x10^9/L	1.00, omitted	0.93, 0.26 – 3.28, 0.91	1.65, 0.79 – 3.47, 0.18	
Leukocytes count <4 x10^9/L	0.43, 0.05 – 3.66, 0.44	0.48, 0.13 – 1.84, 0.29	1.34, 0.57 – 3.11, 0.50	
Hemoglobin <10 g/dL	0.98, 0.39 – 2.46, 0.97	0.64, 0.28 – 1.42, 0.27	**1.87, 1.15 – 3.04, 0.01**	**1.63, 1.03 – 2.75, 0.04**
Peripheral Blast ≥1%	0.68, 0.27 – 1.71, 0.41	0.95, 0.43 – 2.12, 0.90	1.33, 0.80 – 2.25, 0.27	
Total Symptoms Score ≥20	1.16, 0.43 – 3.10, 0.77	0.67, 0.30 – 1.50, 0.33	**1.83, 1.06 – 3.17, 0.03**	**1.61, 1.02 – 2.80, 0.05**
Spleen length >10 cm BCM	0.82, 0.33 – 2.04, 0.67	1.28, 0.58 – 2.85, 0.54	1.55, 0.96 – 2.52, 0.07	
Time from diagnosis to ruxolitinib start >2 years	2.00, 0.78 – 5.15, 0.15	1.79, 0.79 – 4.07, 0.17	0.74, 0.45 – 1.20, 0.22	

Supplemental Table 2: BID, Bis in die; BCM, Below Costal Margin; OR, Odds Ratio; HR, Hazard Ratio; CI, Confidence Interval; DIPSS/MYSEC score was not reported because it was found to be correlated with most variables (hemoglobin <10 g/dL, TSS ≥20, leukocytes count >25 x10^9/L) by Pearson-test.

Supplemental Table 3: Clinical-laboratory characteristics comparison between Older and Transplant-age Cohort, according to driver mutation

	**Overall cohort**			***CALR*-mutated cohort**			***JAK2*-mutated cohort**		
	**Transplant-age (n. 523)**	**Older-age (n. 398)**	**p**	**Transplant-age (n. 82)**	**Older-age (n. 53)**	**p**	**Transplant-age (n. 441)**	**Older-age (n. 345)**	**p**
**Median age, years (range)**	62.5 (24.0-69.9)	75.4 (70.1-92.6)	**<0.001**	60.6 (24.0-69.8)	74.1 (70.0-87.2)	**<0.001**	62.8 (26.5-69.9)	75.8 (70.0-92.6)	<0.001
**Male Sex, n. (%)**	294 (56.2%)	228 (57.3%)	0.80	51 (62.2%)	26 (49.1%)	0.13	243 (55.1%)	202 (58.4%)	0.38
**Primary Myelofibrosis, n. (%)**	263 (50.3%)	203 (51.0%)	0.88	45 (54.9%)	34 (64.2%)	0.29	218 (49.4%)	169 (49.0%)	0.85
**Ruxolitinib starting daily dose, n. (%)**									
10-20 mg	188 (35.9%)	173 (43.5%)	**0.02**	30 (36.6%)	23 (43.4%)	0.38	158 (35.8%)	150 (43.5%)	**0.02**
30-40 mg	335 (64.1%)	225 (56.5%)		52 (63.4%)	30 (56.6%)		283 (64.2%)	195 (56.5%)	
**Lower than prescribing dose, n. (%)**	199 (38.0%)	179 (45.0%)	**0.03**	27 (32.9%)	25 (47.2%)	0.08	172 (39.2%)	154 (44.6%)	0.11
Dose reduction at 6 months, n. (%)	91/319 (28.5%)	77/200 (38.5%)	**0.02**	14/46 (30.4%)	10/29 (34.5%)	0.71	77/273 (28.2%)	67/171 (39.2%)	**0.02**
**Grade of fibrosis < 2, n. (%)**	125/494 (25.3%)	73/380 (19.2%)	**0.04**	12/77 (15.6%)	5/50 (10.0%)	0.37	113/417 (27.1%)	68/330 (20.6%)	**0.05**
**DIPSS[1]/MYSEC-PM[2] risk score, n. (%)**									
Intermediate-1	353 (67.5%)	183 (46.0%)	**<0.001**	52 (63.4%)	20 (37.7%)	**0.004**	301 (68.3%)	164 (47.5%)	**<0.001**
Intermediate-2	142 (27.2%)	162 (40.7%)		25 (30.5%)	22 (41.5%)		117 (26.6%)	140 (40.6%)	
High	28 (5.4%)	53 (13.3%)		5 (6.1%)	11 (20.8%)		23 (5.2%)	41 (11.9%)	
**High Molecular Risk mutation, n. (%)**	87/172 (50.6%)	39/80 (48.8%)	0.79	24/37 (64.9%)	8/14 (57.1%)	0.61	63/135 (46.7%)	31/66 (47.0%)	0.97
**Platelet count, median (range), x 10^9/L**	263.5 (14-1425)	265.5 (26-1887)	0.81	250.5 (53-982)	359 (53 – 1887)	0.06	264.5 (14-1425)	252 (26 – 1632)	0.21
Platelet count < 100 x 10^9/L	48 (9.2%	41 (10.3%)	0.59	11 (13.4%)	4 (7.6%)	0.29	37 (8.4%)	37 (10.7%)	0.28
**Leukocytes, median (range), x 10^9/L,**	10.4 (2.1-80)	12.5 (1.1-155)	**0.003**	8.7 (2.9-71.1)	8.3 (2.1 – 42.9)	0.70	11 (2.1-80)	13.5 (1.1 – 155)	**0.001**
Leukocytes >25 x 10^9/L, n. (%)	70 (13.4%)	72 (18.1%)	0.06	6 (7.3%)	6 (11.3%)	0.43	64 (14.5%)	66 (19.1%)	0.09
Leukocytes <4 x 10^9/L, n. (%)	46 (8.8%)	30 (7.5%)	0.48	8 (9.8%)	5 (9.4%)	0.95	38 (8.6%)	25 (7.2%)	0.47
**Hemoglobin, median (range), g/dL**	11.3 (5.6-18.3)	10.6 (5.7-16.7)	**<0.001**	10.6 (5.6-14.4)	10.0 (6.6 – 14.5)	0.31	11.5 (6.0-18.3)	10.6 (5.7 – 16.7)	**0.001**
Haemoglobin < 10 g/dL, n. (%)	162 (31.0%)	160 (40.2%)	**0.004**	33 (40.2%)	26 (49.1%)	0.31	129 (29.3%)	134 (38.8%)	**0.006**
**Blasts, mean ± SD, %**	0.99 ± 1.7	0.93 ± 1.6	0.93	1.6 ± 2.1	1.5 ± 2.0	0.80	0.9 ± 1.6	0.8 ± 1.5	0.80
Blasts ≥ 1%, n. (%)	192/506 (37.9%)	145/388 (37.4%)	0.86	45/81 (55.6%)	28 (52.8%)	0.94	147/425 (34.6%)	117 (33.9%)	0.97
**Spleen length below costal margin, median (range), cm**	10 (0-35)	10 (0-35)	0.66	9 (0 – 30)	8 (0 – 25)	0.60	10 (0 – 35)	10 (0 – 35)	0.50
Spleen > 10 cm, n. (%)	224/517 (43.3%)	186 (46.7%)	0.26	36/81 (44.4%)	21 (39.6%)	0.58	188/436 (43.1%)	165 (47.8%)	0.15
**Total Symptoms Score, median (range)**	20 (0-100)	20 (0-100)	0.20	20 (0-87)	24 (0 – 80)	**0.01**	20 (0-100)	20 (0 – 100)	0.71
Total Symptoms Score ≥ 20, n. (%)	298/492 (60.6%)	233/372 (62.6%)	0.54	40/76 (52.6%)	34/47 (72.3%)	**0.03**	258/416 (62.0%)	199/325 (61.2%)	0.83
**Years between MF diagnosis and ruxolitinib start, median (range),**	0.8 (0-32.9)	1.0 (0-23.5)	0.30	2.8 (0 – 32.9)	2.0 (0.05 – 22.3)	0.67	0.6 (0 – 28.1)	0.9 (0 – 23.5)	0.13
>2 years, n. (%)	196 (37.5%)	147 (36.9%)	0.83	46 (56.1%)	26 (49.1%)	0.42	150 (34.0%)	121 (35.1%)	0.80
**Spleen response at 6 months, n. (%)**	107/446 (24.0%)	87/327 (26.6%)	0.41	16/68 (23.5%)	8/44 (18.2%)	0.50	91/376 (24.2%)	79/283 (27.9%)	0.26
**Symptoms response at 6 months, n. (%)**	261/391 (66.8%)	192/304 (63.2%)	0.32	31/59 (52.5%)	24/39 (61.5%)	0.38	230/332 (69.3%)	168/265 (63.4%)	0.13
**Any grade Haematological Toxicity at 6 months**									
Overall anaemia, n. (%)	213/468 (45.5%)	208/345 (60.3%)	**<0.001**	41/74 (55.4%)	32/47 (68.1%)	0.17	169/394 (42.9%)	176/298 (59.1%)	**<0.001**
Treatment-emergent anemia, n. (%)	82/322 (25.5%)	84/212 (39.6%)	**0.001**	15/45 (33.3%)	10/25 (40.0%)	0.58	67/277 (24.2%)	74/187 (39.6%)	**<0.001**
Overall thrombocytopenia, n. (%)	93/466 (20.0%)	96/345 (27.8%)	**0.009**	16/73 (21.9%)	9/47 (19.2%)	0.72	77/393 (19.6%)	87/298 (29.2%)	**0.003**
Treatment-emergent thrombocytopenia, n. (%)	68/436 (15.6%)	66/316 (20.9%)	0.06	12/67 (17.9%)	6/45 (13.3%)	0.52	56/369 (15.2%)	60/271 (22.1%)	**0.02**

Supplemental Table 3: 1) Passamonti F, et al. Blood. 2010;116(15):2857-2858; 2) Passamonti F, et al. Leukemia. 2017;31(12):2726-2731
